# Myositis ossificans progressive: case report

**DOI:** 10.11604/pamj.2016.24.264.6670

**Published:** 2016-07-21

**Authors:** Sofia Talbi, Nassira Aradoini, Iman El Mezouar, Fatima Ezzahra Abourazzak, Taoufik Harzy

**Affiliations:** 1 Department of Rheumatology, University Hospital Hassan II, Fes, Morocco

**Keywords:** Myositis ossificans progressive, ossification, ectopic bone

## Abstract

Myositis ossificans progressiva (MOP) is an autosomal dominant disorder. There is a progressive ectopic ossification and skeletal malformation, mainly in the connective tissue of muscle. The diagnosis is based on the clinical findings and radiological demonstration of the skeletal malformations. A 38-year-old female patient was admitted to our department with progressive increase of the thigh. Results of laboratory studies were normal. The radiography of the right thigh showed multiple intramuscular calcifications. Myositis ossificans progressiva should be diagnosed as early as possible and non-invasively, based upon history, clinical and radiological findings. Early and correct diagnosis is fundamental for indication of proper management of the disease.

## Introduction

Myositis ossificans progressiva (MOP) is an autosomal dominant disorder. This disease is very rare with an incidence of less than 1 in 10,000,000 populations. There is a progressive ectopic ossification and skeletal malformation, mainly in the connective tissue of muscle. Muscles most often involved are brachialis, quadriceps femoris and adductor muscles of thigh. The diagnosis is based on the clinical findings and radiological demonstration of the skeletal malformations. Early and correct diagnosis is fundamental for indication of proper management of the disease. We report the case of a 38-year-old woman with clinical and radiological features of MOP.

## Patient and observation

**Figure 1:**
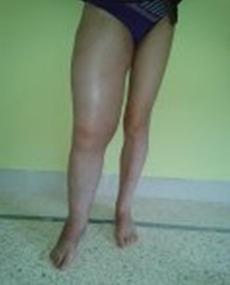
Clinical aspect of thigh

**Figure 2:**
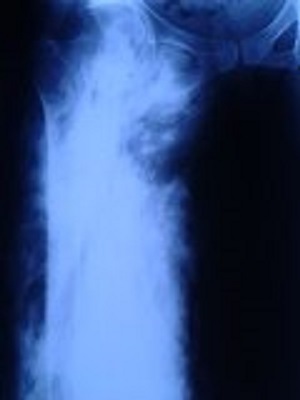
Radiography of the right thigh showed a large calcifications or ectopic bone of soft tissues

A 38 -year-old female patient consulted at our department. She has no family or individual medical history. She has presented 3 years earlier, a progressive increase in thigh volume with functional disability. On the physical examination we have found a painless mass of the right thigh with hard consistency and neovascularization (Figure 1). Results of laboratory studies were normal: erythrocyte sedimentation rate (ESR) C-reactive protein (CRP) and calcium and phosphate levels. The radiography of the right thigh showed a large calcifications or ectopic bone of soft tissues (Figure 2). The magnetic resonance imaging (MRI) of the thigh has shown a significant increase of the right thigh muscles witha high signal intensity on T2- weighted images related tocalcification area. A muscle biopsy was done with immunohistological study and the result was in favor of myositis ossificans. Written informed consent was obtained from the patient’s legal guardian for publication of the case.

## Discussion

Myositis ossificans progressiva is a rare hereditary mesodermal disorder, It is a mutation in chromosome 2 in the bone morphogenetic protein type, receptor ACVRI, with an incidence of less than 1 in 10,000,000 populations affecting all ethnic backgrounds with both sexes equally [1]. More than 95% of cases are sporadic and comes from de novomutation. There is progressive ectopic ossification and characteristics skeletal malformations like malformations of the big toe. Ectopic bone formation occurs in the connective tissue within the muscles, fascia, tendons, ligaments and joint capsules [2]. In our case the woman does not present the malformation of the big toe. This malformation was reported in 79-100% of patients is considered pathognomonic sign [3]. Early clinical manifestation is characterized by soft tissue ossification, especially of the neck and other areas, such as those of the lower limbs and dorsum can also be affected in early clinical status [4]. The disease is characterized by frequent edemas as a result of inflammatory processes that causes ossification and, subsequently, restriction of motility of the affected region which may become painful. The women in the case discussed had no pain but his movements were apparently restricted. The diagnosis depends on the clinical and radiological demonstration of the characteristic skeletal malformation. Routine laboratory tests are usually normal or non-contributive [5]. Moreover, the severity of the disease may be underestimated by X-rays. Magnetic Resonance Imaging has a role in making the diagnosis in the early stage (preosseous stage). The preosseous lesions usually have low signal intensity on T1-weighted images and high signal intensity on T2- weighted images [6]. Bone scintigraphy with 99mTc -MDP may show early ectopic ossification and may also contribute to the evaluation of the progression and the extent of the disease [7]. If the diagnosis of MOP is clear on clinical and radiological grounds calcified nodules biopsy should be avoided because it can result in recurrent ossification of the biopsy site [8]. Exacerbation of MOP may occur in spontaneously or be precipitated by trauma, Such As by surgical operations or intramuscular injections; however they usually appear independently of extrinsic factors, with unpredictable frequency [2]. Differential diagnosis of MOP is obtained with dermatomyositis, tumoral idiopathic calcinosis, idiopathic calcinosis, and calcium metabolism diseases, Osteosarcoma but in this case it is extremely painful with a high level of alkaline phosphatase. Many treatments have been used, Corticosteroids, non-steroidal anti-inflammatory drugs but without results. In vitro chemical studies have shown that bisphosphonates adsorb hydroxyapatite crystals, thus diminishing heterotopic bone formation during the active stage of the disease [9], when there is a severe limitation on movements and gastric intolerance, use of intravenous bisphosphonate may be indicated and may produce a good improvement. However, disease progression remains unchanged.

## Conclusion

Myositis ossificans progressive must diagnosed early and noninvasivel, based on history, clinical and radiological discovery. Early and correct diagnosis is fundamental for indication of proper management of the disease. Exacerbation of the disease may occur spontaneously or be precipitated by trauma, such as by surgical operations or intramuscular injections. If the diagnosis of MOP is clear on clinical and radiological grounds the biopsy of calcified nodules should be avoided.
